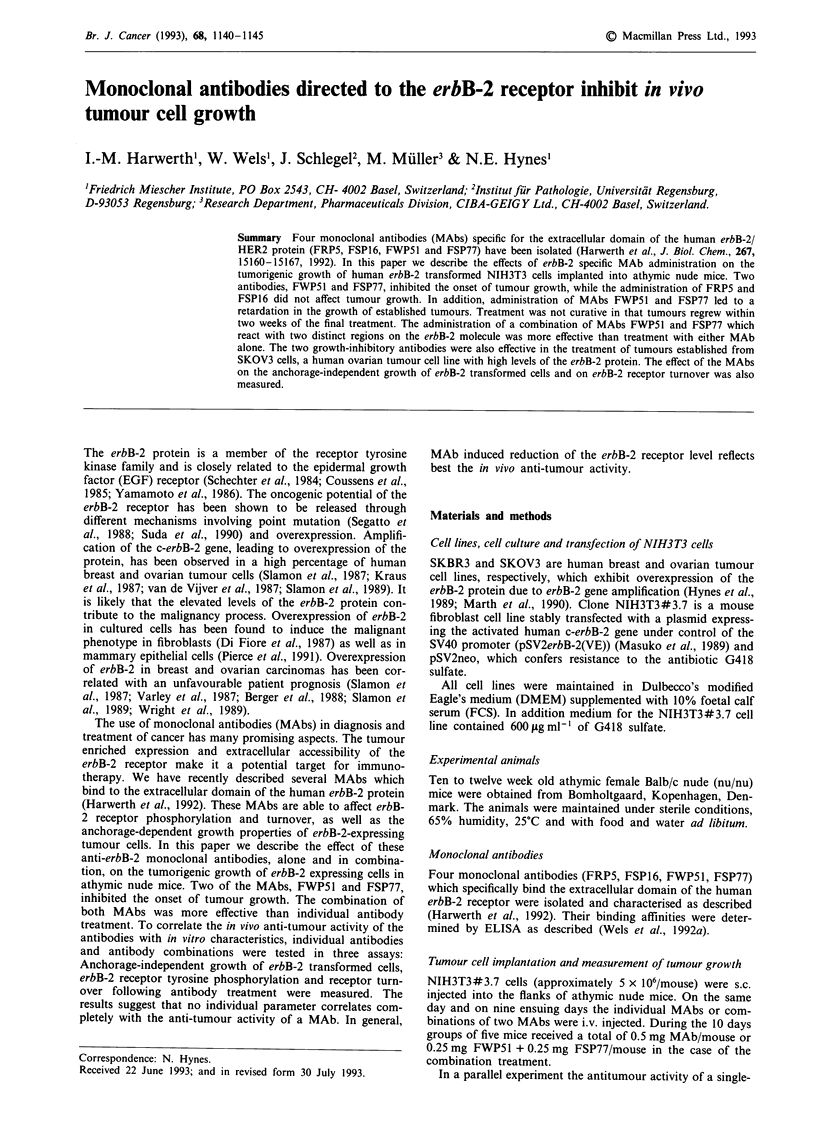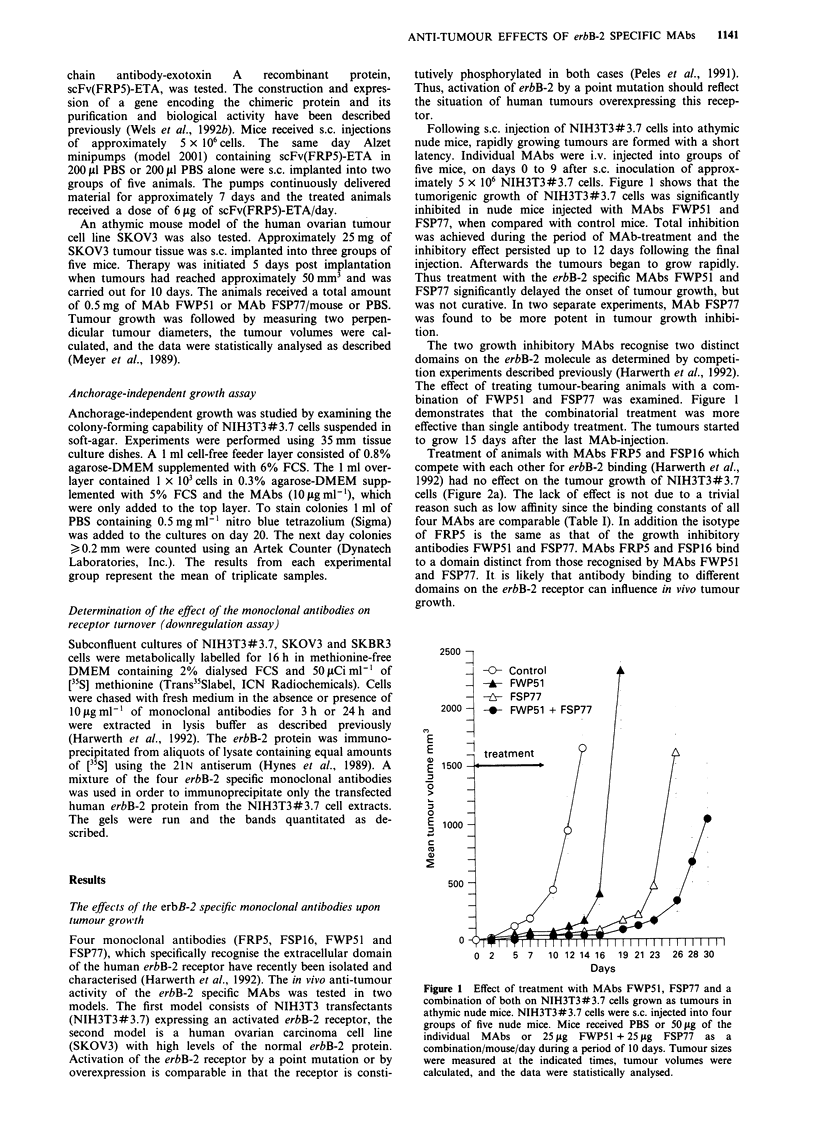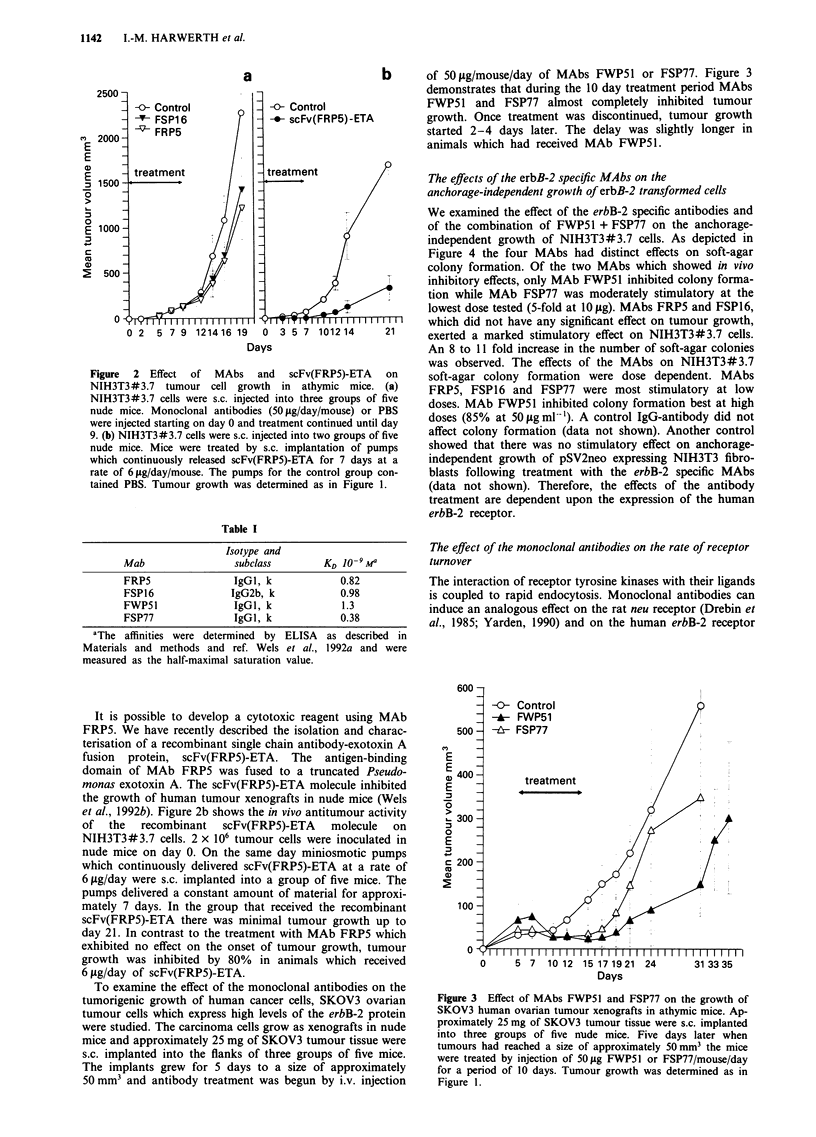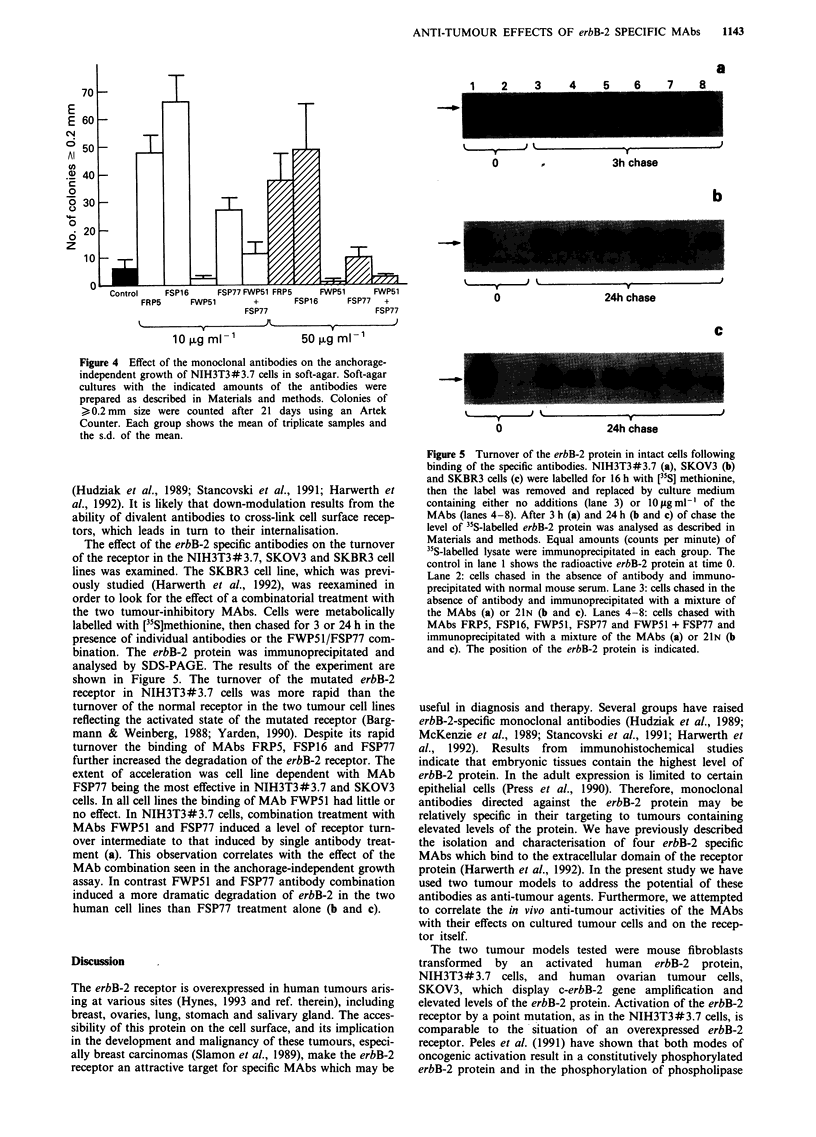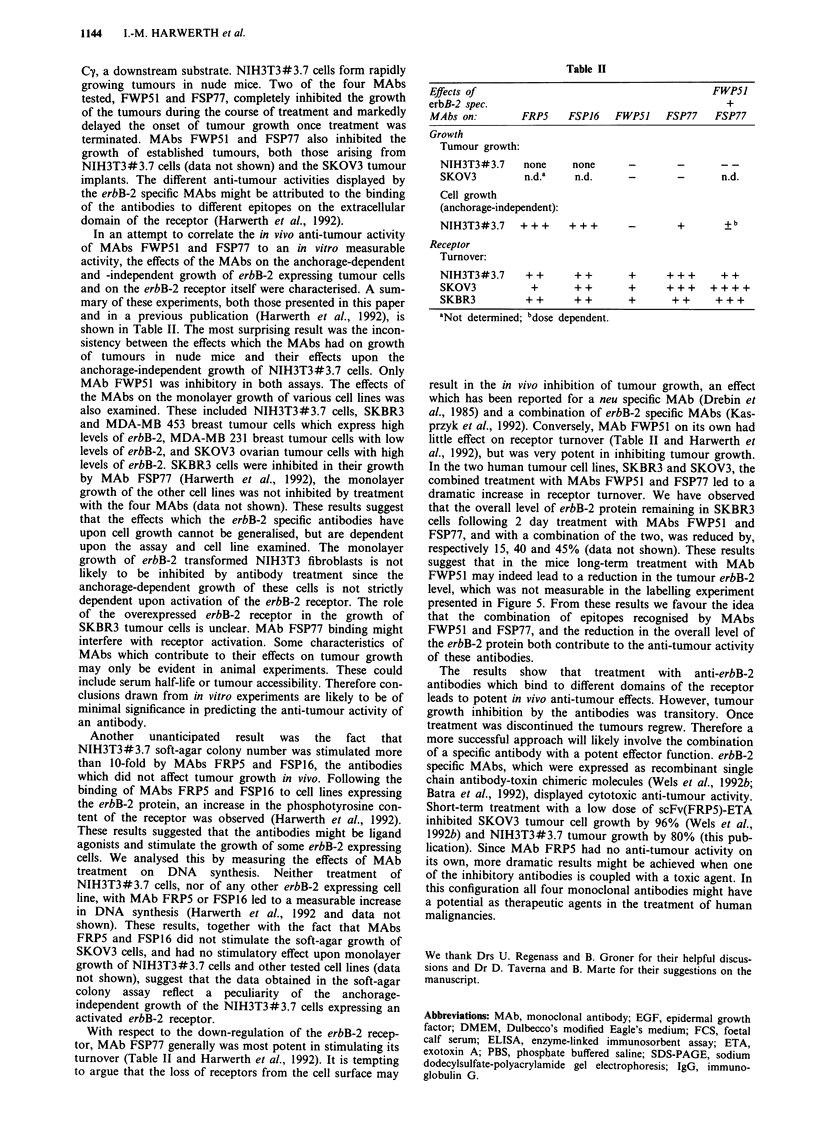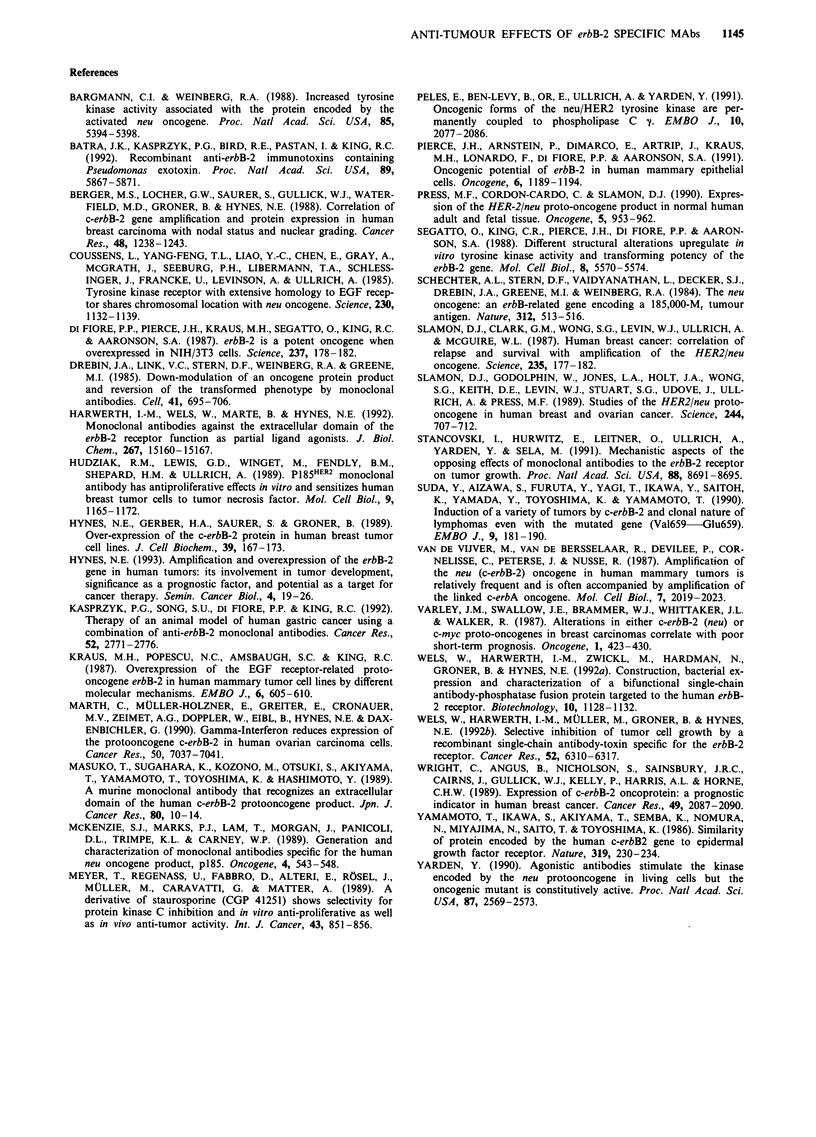# Monoclonal antibodies directed to the erbB-2 receptor inhibit in vivo tumour cell growth.

**DOI:** 10.1038/bjc.1993.494

**Published:** 1993-12

**Authors:** I. M. Harwerth, W. Wels, J. Schlegel, M. Müller, N. E. Hynes

**Affiliations:** Friedrich Miescher Institute, Basel, Switzerland.

## Abstract

**Images:**


Br. J. Cancer (1993), 68, 1140–1145 © Macmillan Press Ltd., 1993

### Monoclonal antibodies directed to the *erb*B-2 receptor inhibit *in vivo* tumour cell growth

I.-M. Harwerth[1], W. Wels[1], J. Schlegel[2], M. Müller[3] & N.E. Hynes[1]

[1]*Friedrich Miescher Institute, PO Box 2543, CH- 4002 Basel, Switzerland;* [2]*Institut für Pathologie, Universität Regensburg, D-93053 Regensburg;* [3]*Research Department, Pharmaceuticals Division, CIBA-GEIGY Ltd., CH-4002 Basel, Switzerland.*

**Summary** Four monoclonal antibodies (MAbs) specific for the extracellular domain of the human *erb*B-2/HER2 protein (FRP5, FSP16, FWP51 and FSP77) have been isolated (Harwerth *et al.*, *J. Biol. Chem.*, **267**, 15160–15167, 1992). In this paper we describe the effects of *erb*B-2 specific MAb administration on the tumorigenic growth of human *erb*B-2 transformed NIH3T3 cells implanted into athymic nude mice. Two antibodies, FWP51 and FSP77, inhibited the onset of tumour growth, while the administration of FRP5 and FSP16 did not affect tumour growth. In addition, administration of MAbs FWP51 and FSP77 led to a retardation in the growth of established tumours. Treatment was not curative in that tumours regrew within two weeks of the final treatment. The administration of a combination of MAbs FWP51 and FSP77 which react with two distinct regions on the *erb*B-2 molecule was more effective than treatment with either MAb alone. The two growth-inhibitory antibodies were also effective in the treatment of tumours established from SKOV3 cells, a human ovarian tumour cell line with high levels of the *erb*B-2 protein. The effect of the MAbs on the anchorage-independent growth of *erb*B-2 transformed cells and on *erb*B-2 receptor turnover was also measured.

The *erb*B-2 protein is a member of the receptor tyrosine kinase family and is closely related to the epidermal growth factor (EGF) receptor (Schechter *et al.*, 1984; Coussens *et al.*, 1985; Yamamoto *et al.*, 1986). The oncogenic potential of the *erb*B-2 receptor has been shown to be released through different mechanisms involving point mutation (Segatto *et al.*, 1988; Suda *et al.*, 1990) and overexpression. Amplification of the c-*erb*B-2 gene, leading to overexpression of the protein, has been observed in a high percentage of human breast and ovarian tumour cells (Slamon *et al.*, 1987; Kraus *et al.*, 1987; van de Vijver *et al.*, 1987; Slamon *et al.*, 1989). It is likely that the elevated levels of the *erb*B-2 protein contribute to the malignancy process. Overexpression of *erb*B-2 in cultured cells has been found to induce the malignant phenotype in fibroblasts (Di Fiore *et al.*, 1987) as well as in mammary epithelial cells (Pierce *et al.*, 1991). Overexpression of *erb*B-2 in breast and ovarian carcinomas has been correlated with an unfavourable patient prognosis (Slamon *et al.*, 1987; Varley *et al.*, 1987; Berger *et al.*, 1988; Slamon *et al.*, 1989; Wright *et al.*, 1989).

The use of monoclonal antibodies (MAbs) in diagnosis and treatment of cancer has many promising aspects. The tumour enriched expression and extracellular accessibility of the *erb*B-2 receptor make it a potential target for immunotherapy. We have recently described several MAbs which bind to the extracellular domain of the human *erb*B-2 protein (Harwerth *et al.*, 1992). These MAbs are able to affect *erb*B-2 receptor phosphorylation and turnover, as well as the anchorage-dependent growth properties of *erb*B-2-expressing tumour cells. In this paper we describe the effect of these anti-*erb*B-2 monoclonal antibodies, alone and in combination, on the tumorigenic growth of *erb*B-2 expressing cells in athymic nude mice. Two of the MAbs, FWP51 and FSP77, inhibited the onset of tumour growth. The combination of both MAbs was more effective than individual antibody treatment. To correlate the *in vivo* anti-tumour activity of the antibodies with *in vitro* characteristics, individual antibodies and antibody combinations were tested in three assays: Anchorage-independent growth of *erb*B-2 transformed cells, *erb*B-2 receptor tyrosine phosphorylation and receptor turnover following antibody treatment were measured. The results suggest that no individual parameter correlates completely with the anti-tumour activity of a MAb. In general, MAb induced reduction of the *erb*B-2 receptor level reflects best the *in vivo* anti-tumour activity.

Correspondence: N. Hynes.
Received 22 June 1993; and in revised form 30 July 1993.

### Materials and methods

### *Cell lines, cell culture and transfection of NIH3T3 cells*

SKBR3 and SKOV3 are human breast and ovarian tumour cell lines, respectively, which exhibit overexpression of the *erb*B-2 protein due to *erb*B-2 gene amplification (Hynes *et al.*, 1989; Marth *et al.*, 1990). Clone NIH3T3#3.7 is a mouse fibroblast cell line stably transfected with a plasmid expressing the activated human c-*erb*B-2 gene under control of the SV40 promoter (pSV2*erb*B-2(VE)) (Masuko *et al.*, 1989) and pSV2neo, which confers resistance to the antibiotic G418 sulfate.

All cell lines were maintained in Dulbecco's modified Eagle's medium (DMEM) supplemented with 10% foetal calf serum (FCS). In addition medium for the NIH3T3#3.7 cell line contained 600 $\mu g\ ml^{-1}$ of G418 sulfate.

### *Experimental animals*

Ten to twelve week old athymic female Balb/c nude (nu/nu) mice were obtained from Bomholtgaard, Kopenhagen, Denmark. The animals were maintained under sterile conditions, 65% humidity, 25°C and with food and water *ad libitum.*

### *Monoclonal antibodies*

Four monoclonal antibodies (FRP5, FSP16, FWP51, FSP77) which specifically bind the extracellular domain of the human *erb*B-2 receptor were isolated and characterised as described (Harwerth *et al.*, 1992). Their binding affinities were determined by ELISA as described (Wels *et al.*, 1992*a*).

### *Tumour cell implantation and measurement of tumour growth*

NIH3T3#3.7 cells (approximately $5 \times 10^6$/mouse) were s.c. injected into the flanks of athymic nude mice. On the same day and on nine ensuing days the individual MAbs or combinations of two MAbs were i.v. injected. During the 10 days groups of five mice received a total of 0.5 mg MAb/mouse or 0.25 mg FWP51 + 0.25 mg FSP77/mouse in the case of the combination treatment.

In a parallel experiment the antitumour activity of a singlechain antibody-exotoxin A recombinant protein, scFv(FRP5)-ETA, was tested. The construction and expression of a gene encoding the chimeric protein and its purification and biological activity have been described previously (Wels *et al.*, 1992*b*). Mice received s.c. injections of approximately $5 \times 10^6$ cells. The same day Alzet minipumps (model 2001) containing scFv(FRP5)-ETA in 200 µl PBS or 200 µl PBS alone were s.c. implanted into two groups of five animals. The pumps continuously delivered material for approximately 7 days and the treated animals received a dose of 6 µg of scFv(FRP5)-ETA/day.

An athymic mouse model of the human ovarian tumour cell line SKOV3 was also tested. Approximately 25 mg of SKOV3 tumour tissue was s.c. implanted into three groups of five mice. Therapy was initiated 5 days post implantation when tumours had reached approximately 50 mm$^3$ and was carried out for 10 days. The animals received a total amount of 0.5 mg of MAb FWP51 or MAb FSP77/mouse or PBS. Tumour growth was followed by measuring two perpendicular tumour diameters, the tumour volumes were calculated, and the data were statistically analysed as described (Meyer *et al.*, 1989).

*Anchorage-independent growth assay*

Anchorage-independent growth was studied by examining the colony-forming capability of NIH3T3#3.7 cells suspended in soft-agar. Experiments were performed using 35 mm tissue culture dishes. A 1 ml cell-free feeder layer consisted of 0.8% agarose-DMEM supplemented with 6% FCS. The 1 ml overlayer contained $1 \times 10^3$ cells in 0.3% agarose-DMEM supplemented with 5% FCS and the MAbs (10 µg ml$^{-1}$), which were only added to the top layer. To stain colonies 1 ml of PBS containing 0.5 mg ml$^{-1}$ nitro blue tetrazolium (Sigma) was added to the cultures on day 20. The next day colonies $\geqslant$0.2 mm were counted using an Artek Counter (Dynatech Laboratories, Inc.). The results from each experimental group represent the mean of triplicate samples.

*Determination of the effect of the monoclonal antibodies on receptor turnover (downregulation assay)*

Subconfluent cultures of NIH3T3#3.7, SKOV3 and SKBR3 cells were metabolically labelled for 16 h in methionine-free DMEM containing 2% dialysed FCS and 50 µCi ml$^{-1}$ of [$^{35}$S] methionine (Trans$^{35}$Slabel, ICN Radiochemicals). Cells were chased with fresh medium in the absence or presence of 10 µg ml$^{-1}$ of monoclonal antibodies for 3 h or 24 h and were extracted in lysis buffer as described previously (Harwerth *et al.*, 1992). The *erb*B-2 protein was immunoprecipitated from aliquots of lysate containing equal amounts of [$^{35}$S] using the 21N antiserum (Hynes *et al.*, 1989). A mixture of the four *erb*B-2 specific monoclonal antibodies was used in order to immunoprecipitate only the transfected human *erb*B-2 protein from the NIH3T3#3.7 cell extracts. The gels were run and the bands quantitated as described.

### Results

*The effects of the* erbB-2 *specific monoclonal antibodies upon tumour growth*

Four monoclonal antibodies (FRP5, FSP16, FWP51 and FSP77), which specifically recognise the extracellular domain of the human *erb*B-2 receptor have recently been isolated and characterised (Harwerth *et al.*, 1992). The *in vivo* anti-tumour activity of the *erb*B-2 specific MAbs was tested in two models. The first model consists of NIH3T3 transfectants (NIH3T3#3.7) expressing an activated *erb*B-2 receptor, the second model is a human ovarian carcinoma cell line (SKOV3) with high levels of the normal *erb*B-2 protein. Activation of the *erb*B-2 receptor by a point mutation or by overexpression is comparable in that the receptor is constitutively phosphorylated in both cases (Peles *et al.*, 1991). Thus, activation of *erb*B-2 by a point mutation should reflect the situation of human tumours overexpressing this receptor.

Following s.c. injection of NIH3T3#3.7 cells into athymic nude mice, rapidly growing tumours are formed with a short latency. Individual MAbs were i.v. injected into groups of five mice, on days 0 to 9 after s.c. inoculation of approximately $5 \times 10^6$ NIH3T3#3.7 cells. Figure 1 shows that the tumorigenic growth of NIH3T3#3.7 cells was significantly inhibited in nude mice injected with MAbs FWP51 and FSP77, when compared with control mice. Total inhibition was achieved during the period of MAb-treatment and the inhibitory effect persisted up to 12 days following the final injection. Afterwards the tumours began to grow rapidly. Thus treatment with the *erb*B-2 specific MAbs FWP51 and FSP77 significantly delayed the onset of tumour growth, but was not curative. In two separate experiments, MAb FSP77 was found to be more potent in tumour growth inhibition.

The two growth inhibitory MAbs recognise two distinct domains on the *erb*B-2 molecule as determined by competition experiments described previously (Harwerth *et al.*, 1992). The effect of treating tumour-bearing animals with a combination of FWP51 and FSP77 was examined. Figure 1 demonstrates that the combinatorial treatment was more effective than single antibody treatment. The tumours started to grow 15 days after the last MAb-injection.

Treatment of animals with MAbs FRP5 and FSP16 which compete with each other for *erb*B-2 binding (Harwerth *et al.*, 1992) had no effect on the tumour growth of NIH3T3#3.7 cells (Figure 2a). The lack of effect is not due to a trivial reason such as low affinity since the binding constants of all four MAbs are comparable (Table I). In addition the isotype of FRP5 is the same as that of the growth inhibitory antibodies FWP51 and FSP77. MAbs FRP5 and FSP16 bind to a domain distinct from those recognised by MAbs FWP51 and FSP77. It is likely that antibody binding to different domains on the *erb*B-2 receptor can influence *in vivo* tumour growth.

**Figure 1** Effect of treatment with MAbs FWP51, FSP77 and a combination of both on NIH3T3#3.7 cells grown as tumours in athymic nude mice. NIH3T3#3.7 cells were s.c. injected into four groups of five nude mice. Mice received PBS or 50 µg of the individual MAbs or 25 µg FWP51 + 25 µg FSP77 as a combination/mouse/day during a period of 10 days. Tumour sizes were measured at the indicated times, tumour volumes were calculated, and the data were statistically analysed.

**Figure 2** Effect of MAbs and scFv(FRP5)-ETA on NIH3T3#3.7 tumour cell growth in athymic mice. (**a**) NIH3T3#3.7 cells were s.c. injected into three groups of five nude mice. Monoclonal antibodies (50 μg/day/mouse) or PBS were injected starting on day 0 and treatment continued until day 9. (**b**) NIH3T3#3.7 cells were s.c. injected into two groups of five nude mice. Mice were treated by s.c. implantation of pumps which continuously released scFv(FRP5)-ETA for 7 days at a rate of 6 μg/day/mouse. The pumps for the control group contained PBS. Tumour growth was determined as in Figure 1.

**Table I**

| *Mab* | *Isotype and subclass* | $K_D$ $10^{-9}$ $M^a$ |
|---|---|---|
| FRP5 | IgG1, k | 0.82 |
| FSP16 | IgG2b, k | 0.98 |
| FWP51 | IgG1, k | 1.3 |
| FSP77 | IgG1, k | 0.38 |

[a]The affinities were determined by ELISA as described in Materials and methods and ref. Wels *et al.*, 1992*a* and were measured as the half-maximal saturation value.

It is possible to develop a cytotoxic reagent using MAb FRP5. We have recently described the isolation and characterisation of a recombinant single chain antibody-exotoxin A fusion protein, scFv(FRP5)-ETA. The antigen-binding domain of MAb FRP5 was fused to a truncated *Pseudomonas* exotoxin A. The scFv(FRP5)-ETA molecule inhibited the growth of human tumour xenografts in nude mice (Wels *et al.*, 1992*b*). Figure 2b shows the *in vivo* antitumour activity of the recombinant scFv(FRP5)-ETA molecule on NIH3T3#3.7 cells. $2 \times 10^6$ tumour cells were inoculated in nude mice on day 0. On the same day miniosmotic pumps which continuously delivered scFv(FRP5)-ETA at a rate of 6 μg/day were s.c. implanted into a group of five mice. The pumps delivered a constant amount of material for approximately 7 days. In the group that received the recombinant scFv(FRP5)-ETA there was minimal tumour growth up to day 21. In contrast to the treatment with MAb FRP5 which exhibited no effect on the onset of tumour growth, tumour growth was inhibited by 80% in animals which received 6 μg/day of scFv(FRP5)-ETA.

To examine the effect of the monoclonal antibodies on the tumorigenic growth of human cancer cells, SKOV3 ovarian tumour cells which express high levels of the *erb*B-2 protein were studied. The carcinoma cells grow as xenografts in nude mice and approximately 25 mg of SKOV3 tumour tissue were s.c. implanted into the flanks of three groups of five mice. The implants grew for 5 days to a size of approximately 50 $mm^3$ and antibody treatment was begun by i.v. injection of 50 μg/mouse/day of MAbs FWP51 or FSP77. Figure 3 demonstrates that during the 10 day treatment period MAbs FWP51 and FSP77 almost completely inhibited tumour growth. Once treatment was discontinued, tumour growth started 2–4 days later. The delay was slightly longer in animals which had received MAb FWP51.

*The effects of the* erb*B-2 specific MAbs on the anchorage-independent growth of* erb*B-2 transformed cells*

We examined the effect of the *erb*B-2 specific antibodies and of the combination of FWP51 + FSP77 on the anchorage-independent growth of NIH3T3#3.7 cells. As depicted in Figure 4 the four MAbs had distinct effects on soft-agar colony formation. Of the two MAbs which showed *in vivo* inhibitory effects, only MAb FWP51 inhibited colony formation while MAb FSP77 was moderately stimulatory at the lowest dose tested (5-fold at 10 μg). MAbs FRP5 and FSP16, which did not have any significant effect on tumour growth, exerted a marked stimulatory effect on NIH3T3#3.7 cells. An 8 to 11 fold increase in the number of soft-agar colonies was observed. The effects of the MAbs on NIH3T3#3.7 soft-agar colony formation were dose dependent. MAbs FRP5, FSP16 and FSP77 were most stimulatory at low doses. MAb FWP51 inhibited colony formation best at high doses (85% at 50 μg $ml^{-1}$). A control IgG-antibody did not affect colony formation (data not shown). Another control showed that there was no stimulatory effect on anchorage-independent growth of pSV2neo expressing NIH3T3 fibroblasts following treatment with the *erb*B-2 specific MAbs (data not shown). Therefore, the effects of the antibody treatment are dependent upon the expression of the human *erb*B-2 receptor.

*The effect of the monoclonal antibodies on the rate of receptor turnover*

The interaction of receptor tyrosine kinases with their ligands is coupled to rapid endocytosis. Monoclonal antibodies can induce an analogous effect on the rat *neu* receptor (Drebin *et al.*, 1985; Yarden, 1990) and on the human *erb*B-2 receptor

**Figure 3** Effect of MAbs FWP51 and FSP77 on the growth of SKOV3 human ovarian tumour xenografts in athymic mice. Approximately 25 mg of SKOV3 tumour tissue were s.c. implanted into three groups of five nude mice. Five days later when tumours had reached a size of approximately 50 $mm^3$ the mice were treated by injection of 50 μg FWP51 or FSP77/mouse/day for a period of 10 days. Tumour growth was determined as in Figure 1.

**Figure 4** Effect of the monoclonal antibodies on the anchorage-independent growth of NIH3T3#3.7 cells in soft-agar. Soft-agar cultures with the indicated amounts of the antibodies were prepared as described in Materials and methods. Colonies of $\geqslant 0.2$ mm size were counted after 21 days using an Artek Counter. Each group shows the mean of triplicate samples and the s.d. of the mean.

**Figure 5** Turnover of the *erb*B-2 protein in intact cells following binding of the specific antibodies. NIH3T3#3.7 (**a**), SKOV3 (**b**) and SKBR3 cells (**c**) were labelled for 16 h with [$^{35}$S] methionine, then the label was removed and replaced by culture medium containing either no additions (lane 3) or 10 μg ml$^{-1}$ of the MAbs (lanes 4–8). After 3 h (**a**) and 24 h (**b** and **c**) of chase the level of $^{35}$S-labelled *erb*B-2 protein was analysed as described in Materials and methods. Equal amounts (counts per minute) of $^{35}$S-labelled lysate were immunoprecipitated in each group. The control in lane 1 shows the radioactive *erb*B-2 protein at time 0. Lane 2: cells chased in the absence of antibody and immunoprecipitated with normal mouse serum. Lane 3: cells chased in the absence of antibody and immunoprecipitated with a mixture of the MAbs (**a**) or 21N (**b** and **c**). Lanes 4–8: cells chased with MAbs FRP5, FSP16, FWP51, FSP77 and FWP51 + FSP77 and immunoprecipitated with a mixture of the MAbs (**a**) or 21N (**b** and **c**). The position of the *erb*B-2 protein is indicated.

(Hudziak *et al.*, 1989; Stancovski *et al.*, 1991; Harwerth *et al.*, 1992). It is likely that down-modulation results from the ability of divalent antibodies to cross-link cell surface receptors, which leads in turn to their internalisation.

The effect of the *erb*B-2 specific antibodies on the turnover of the receptor in the NIH3T3#3.7, SKOV3 and SKBR3 cell lines was examined. The SKBR3 cell line, which was previously studied (Harwerth *et al.*, 1992), was reexamined in order to look for the effect of a combinatorial treatment with the two tumour-inhibitory MAbs. Cells were metabolically labelled with [$^{35}$S]methionine, then chased for 3 or 24 h in the presence of individual antibodies or the FWP51/FSP77 combination. The *erb*B-2 protein was immunoprecipitated and analysed by SDS-PAGE. The results of the experiment are shown in Figure 5. The turnover of the mutated *erb*B-2 receptor in NIH3T3#3.7 cells was more rapid than the turnover of the normal receptor in the two tumour cell lines reflecting the activated state of the mutated receptor (Bargmann & Weinberg, 1988; Yarden, 1990). Despite its rapid turnover the binding of MAbs FRP5, FSP16 and FSP77 further increased the degradation of the *erb*B-2 receptor. The extent of acceleration was cell line dependent with MAb FSP77 being the most effective in NIH3T3#3.7 and SKOV3 cells. In all cell lines the binding of MAb FWP51 had little or no effect. In NIH3T3#3.7 cells, combination treatment with MAbs FWP51 and FSP77 induced a level of receptor turnover intermediate to that induced by single antibody treatment (**a**). This observation correlates with the effect of the MAb combination seen in the anchorage-independent growth assay. In contrast FWP51 and FSP77 antibody combination induced a more dramatic degradation of *erb*B-2 in the two human cell lines than FSP77 treatment alone (**b** and **c**).

### Discussion

The *erb*B-2 receptor is overexpressed in human tumours arising at various sites (Hynes, 1993 and ref. therein), including breast, ovaries, lung, stomach and salivary gland. The accessibility of this protein on the cell surface, and its implication in the development and malignancy of these tumours, especially breast carcinomas (Slamon *et al.*, 1989), make the *erb*B-2 receptor an attractive target for specific MAbs which may be useful in diagnosis and therapy. Several groups have raised *erb*B-2-specific monoclonal antibodies (Hudziak *et al.*, 1989; McKenzie *et al.*, 1989; Stancovski *et al.*, 1991; Harwerth *et al.*, 1992). Results from immunohistochemical studies indicate that embryonic tissues contain the highest level of *erb*B-2 protein. In the adult expression is limited to certain epithelial cells (Press *et al.*, 1990). Therefore, monoclonal antibodies directed against the *erb*B-2 protein may be relatively specific in their targeting to tumours containing elevated levels of the protein. We have previously described the isolation and characterisation of four *erb*B-2 specific MAbs which bind to the extracellular domain of the receptor protein (Harwerth *et al.*, 1992). In the present study we have used two tumour models to address the potential of these antibodies as anti-tumour agents. Furthermore, we attempted to correlate the *in vivo* anti-tumour activities of the MAbs with their effects on cultured tumour cells and on the receptor itself.

The two tumour models tested were mouse fibroblasts transformed by an activated human *erb*B-2 protein, NIH3T3#3.7 cells, and human ovarian tumour cells, SKOV3, which display c-*erb*B-2 gene amplification and elevated levels of the *erb*B-2 protein. Activation of the *erb*B-2 receptor by a point mutation, as in the NIH3T3#3.7 cells, is comparable to the situation of an overexpressed *erb*B-2 receptor. Peles *et al.* (1991) have shown that both modes of oncogenic activation result in a constitutively phosphorylated *erb*B-2 protein and in the phosphorylation of phospholipase

Cγ, a downstream substrate. NIH3T3#3.7 cells form rapidly growing tumours in nude mice. Two of the four MAbs tested, FWP51 and FSP77, completely inhibited the growth of the tumours during the course of treatment and markedly delayed the onset of tumour growth once treatment was terminated. MAbs FWP51 and FSP77 also inhibited the growth of established tumours, both those arising from NIH3T3#3.7 cells (data not shown) and the SKOV3 tumour implants. The different anti-tumour activities displayed by the *erb*B-2 specific MAbs might be attributed to the binding of the antibodies to different epitopes on the extracellular domain of the receptor (Harwerth *et al.*, 1992).

In an attempt to correlate the *in vivo* anti-tumour activity of MAbs FWP51 and FSP77 to an *in vitro* measurable activity, the effects of the MAbs on the anchorage-dependent and -independent growth of *erb*B-2 expressing tumour cells and on the *erb*B-2 receptor itself were characterised. A summary of these experiments, both those presented in this paper and in a previous publication (Harwerth *et al.*, 1992), is shown in Table II. The most surprising result was the inconsistency between the effects which the MAbs had on growth of tumours in nude mice and their effects upon the anchorage-independent growth of NIH3T3#3.7 cells. Only MAb FWP51 was inhibitory in both assays. The effects of the MAbs on the monolayer growth of various cell lines was also examined. These included NIH3T3#3.7 cells, SKBR3 and MDA-MB 453 breast tumour cells which express high levels of *erb*B-2, MDA-MB 231 breast tumour cells with low levels of *erb*B-2, and SKOV3 ovarian tumour cells with high levels of *erb*B-2. SKBR3 cells were inhibited in their growth by MAb FSP77 (Harwerth *et al.*, 1992), the monolayer growth of the other cell lines was not inhibited by treatment with the four MAbs (data not shown). These results suggest that the effects which the *erb*B-2 specific antibodies have upon cell growth cannot be generalised, but are dependent upon the assay and cell line examined. The monolayer growth of *erb*B-2 transformed NIH3T3 fibroblasts is not likely to be inhibited by antibody treatment since the anchorage-dependent growth of these cells is not strictly dependent upon activation of the *erb*B-2 receptor. The role of the overexpressed *erb*B-2 receptor in the growth of SKBR3 tumour cells is unclear. MAb FSP77 binding might interfere with receptor activation. Some characteristics of MAbs which contribute to their effects on tumour growth may only be evident in animal experiments. These could include serum half-life or tumour accessibility. Therefore conclusions drawn from *in vitro* experiments are likely to be of minimal significance in predicting the anti-tumour activity of an antibody.

Another unanticipated result was the fact that NIH3T3#3.7 soft-agar colony number was stimulated more than 10-fold by MAbs FRP5 and FSP16, the antibodies which did not affect tumour growth *in vivo*. Following the binding of MAbs FRP5 and FSP16 to cell lines expressing the *erb*B-2 protein, an increase in the phosphotyrosine content of the receptor was observed (Harwerth *et al.*, 1992). These results suggested that the antibodies might be ligand agonists and stimulate the growth of some *erb*B-2 expressing cells. We analysed this by measuring the effects of MAb treatment on DNA synthesis. Neither treatment of NIH3T3#3.7 cells, nor of any other *erb*B-2 expressing cell line, with MAb FRP5 or FSP16 led to a measurable increase in DNA synthesis (Harwerth *et al.*, 1992 and data not shown). These results, together with the fact that MAbs FRP5 and FSP16 did not stimulate the soft-agar growth of SKOV3 cells, and had no stimulatory effect upon monolayer growth of NIH3T3#3.7 cells and other tested cell lines (data not shown), suggest that the data obtained in the soft-agar colony assay reflect a peculiarity of the anchorage-independent growth of the NIH3T3#3.7 cells expressing an activated *erb*B-2 receptor.

With respect to the down-regulation of the *erb*B-2 receptor, MAb FSP77 generally was most potent in stimulating its turnover (Table II and Harwerth *et al.*, 1992). It is tempting to argue that the loss of receptors from the cell surface may

**Table II**

| *Effects of erbB-2 spec. MAbs on:* | *FRP5* | *FSP16* | *FWP51* | *FSP77* | *FWP51 + FSP77* |
|---|---|---|---|---|---|
| *Growth* | | | | | |
| Tumour growth: | | | | | |
| NIH3T3#3.7 | none | none | – | – | – – |
| SKOV3 | n.d.[a] | n.d. | – | – | n.d. |
| Cell growth (anchorage-independent): | | | | | |
| NIH3T3#3.7 | + + + | + + + | – | + | ±[b] |
| *Receptor* | | | | | |
| Turnover: | | | | | |
| NIH3T3#3.7 | + + | + + | + | + + + | + + |
| SKOV3 | + | + + | + | + + + | + + + + |
| SKBR3 | + + | + + | + | + + | + + + |

[a]Not determined; [b]dose dependent.

result in the *in vivo* inhibition of tumour growth, an effect which has been reported for a *neu* specific MAb (Drebin *et al.*, 1985) and a combination of *erb*B-2 specific MAbs (Kasprzyk *et al.*, 1992). Conversely, MAb FWP51 on its own had little effect on receptor turnover (Table II and Harwerth *et al.*, 1992), but was very potent in inhibiting tumour growth. In the two human tumour cell lines, SKBR3 and SKOV3, the combined treatment with MAbs FWP51 and FSP77 led to a dramatic increase in receptor turnover. We have observed that the overall level of *erb*B-2 protein remaining in SKBR3 cells following 2 day treatment with MAbs FWP51 and FSP77, and with a combination of the two, was reduced by, respectively 15, 40 and 45% (data not shown). These results suggest that in the mice long-term treatment with MAb FWP51 may indeed lead to a reduction in the tumour *erb*B-2 level, which was not measurable in the labelling experiment presented in Figure 5. From these results we favour the idea that the combination of epitopes recognised by MAbs FWP51 and FSP77, and the reduction in the overall level of the *erb*B-2 protein both contribute to the anti-tumour activity of these antibodies.

The results show that treatment with anti-*erb*B-2 antibodies which bind to different domains of the receptor leads to potent *in vivo* anti-tumour effects. However, tumour growth inhibition by the antibodies was transitory. Once treatment was discontinued the tumours regrew. Therefore a more successful approach will likely involve the combination of a specific antibody with a potent effector function. *erb*B-2 specific MAbs, which were expressed as recombinant single chain antibody-toxin chimeric molecules (Wels *et al.*, 1992*b*; Batra *et al.*, 1992), displayed cytotoxic anti-tumour activity. Short-term treatment with a low dose of scFv(FRP5)-ETA inhibited SKOV3 tumour cell growth by 96% (Wels *et al.*, 1992*b*) and NIH3T3#3.7 tumour growth by 80% (this publication). Since MAb FRP5 had no anti-tumour activity on its own, more dramatic results might be achieved when one of the inhibitory antibodies is coupled with a toxic agent. In this configuration all four monoclonal antibodies might have a potential as therapeutic agents in the treatment of human malignancies.

We thank Drs U. Regenass and B. Groner for their helpful discussions and Dr D. Taverna and B. Marte for their suggestions on the manuscript.

**Abbreviations:** MAb, monoclonal antibody; EGF, epidermal growth factor; DMEM, Dulbecco's modified Eagle's medium; FCS, foetal calf serum; ELISA, enzyme-linked immunosorbent assay; ETA, exotoxin A; PBS, phosphate buffered saline; SDS-PAGE, sodium dodecylsulfate-polyacrylamide gel electrophoresis; IgG, immunoglobulin G.

### References

BARGMANN, C.I. & WEINBERG, R.A. (1988). Increased tyrosine kinase activity associated with the protein encoded by the activated *neu* oncogene. *Proc. Natl Acad. Sci. USA*, **85**, 5394–5398.

BATRA, J.K., KASPRZYK, P.G., BIRD, R.E., PASTAN, I. & KING, R.C. (1992). Recombinant anti-*erb*B-2 immunotoxins containing *Pseudomonas* exotoxin. *Proc. Natl Acad. Sci. USA*, **89**, 5867–5871.

BERGER, M.S., LOCHER, G.W., SAURER, S., GULLICK, W.J., WATERFIELD, M.D., GRONER, B. & HYNES, N.E. (1988). Correlation of c-*erb*B-2 gene amplification and protein expression in human breast carcinoma with nodal status and nuclear grading. *Cancer Res.*, **48**, 1238–1243.

COUSSENS, L., YANG-FENG, T.L., LIAO, Y.-C., CHEN, E., GRAY, A., MCGRATH, J., SEEBURG, P.H., LIBERMANN, T.A., SCHLESSINGER, J., FRANCKE, U., LEVINSON, A. & ULLRICH, A. (1985). Tyrosine kinase receptor with extensive homology to EGF receptor shares chromosomal location with *neu* oncogene. *Science*, **230**, 1132–1139.

DI FIORE, P.P., PIERCE, J.H., KRAUS, M.H., SEGATTO, O., KING, R.C. & AARONSON, S.A. (1987). *erb*B-2 is a potent oncogene when overexpressed in NIH/3T3 cells. *Science*, **237**, 178–182.

DREBIN, J.A., LINK, V.C., STERN, D.F., WEINBERG, R.A. & GREENE, M.I. (1985). Down-modulation of an oncogene protein product and reversion of the transformed phenotype by monoclonal antibodies. *Cell*, **41**, 695–706.

HARWERTH, I.-M., WELS, W., MARTE, B. & HYNES, N.E. (1992). Monoclonal antibodies against the extracellular domain of the *erb*B-2 receptor function as partial ligand agonists. *J. Biol. Chem.*, **267**, 15160–15167.

HUDZIAK, R.M., LEWIS, G.D., WINGET, M., FENDLY, B.M., SHEPARD, H.M. & ULLRICH, A. (1989). $P185^{HER2}$ monoclonal antibody has antiproliferative effects *in vitro* and sensitizes human breast tumor cells to tumor necrosis factor. *Mol. Cell Biol.*, **9**, 1165–1172.

HYNES, N.E., GERBER, H.A., SAURER, S. & GRONER, B. (1989). Over-expression of the c-*erb*B-2 protein in human breast tumor cell lines. *J. Cell Biochem.*, **39**, 167–173.

HYNES, N.E. (1993). Amplification and overexpression of the *erb*B-2 gene in human tumors: its involvement in tumor development, significance as a prognostic factor, and potential as a target for cancer therapy. *Semin. Cancer Biol.*, **4**, 19–26.

KASPRZYK, P.G., SONG, S.U., DI FIORE, P.P. & KING, R.C. (1992). Therapy of an animal model of human gastric cancer using a combination of anti-*erb*B-2 monoclonal antibodies. *Cancer Res.*, **52**, 2771–2776.

KRAUS, M.H., POPESCU, N.C., AMSBAUGH, S.C. & KING, R.C. (1987). Overexpression of the EGF receptor-related proto-oncogene *erb*B-2 in human mammary tumor cell lines by different molecular mechanisms. *EMBO J.*, **6**, 605–610.

MARTH, C., MÜLLER-HOLZNER, E., GREITER, E., CRONAUER, M.V., ZEIMET, A.G., DOPPLER, W., EIBL, B., HYNES, N.E. & DAXENBICHLER, G. (1990). Gamma-Interferon reduces expression of the protooncogene c-*erb*B-2 in human ovarian carcinoma cells. *Cancer Res.*, 50, 7037–7041.

MASUKO, T., SUGAHARA, K., KOZONO, M., OTSUKI, S., AKIYAMA, T., YAMAMOTO, T., TOYOSHIMA, K. & HASHIMOTO, Y. (1989). A murine monoclonal antibody that recognizes an extracellular domain of the human c-*erb*B-2 protooncogene product. *Jpn. J. Cancer Res.*, **80**, 10–14.

MCKENZIE, S.J., MARKS, P.J., LAM, T., MORGAN, J., PANICOLI, D.L., TRIMPE, K.L. & CARNEY, W.P. (1989). Generation and characterization of monoclonal antibodies specific for the human *neu* oncogene product, p185. *Oncogene*, **4**, 543–548.

MEYER, T., REGENASS, U., FABBRO, D., ALTERI, E., RÖSEL, J., MÜLLER, M., CARAVATTI, G. & MATTER, A. (1989). A derivative of staurosporine (CGP 41251) shows selectivity for protein kinase C inhibition and *in vitro* anti-proliferative as well as *in vivo* anti-tumor activity. *Int. J. Cancer*, **43**, 851–856.

PELES, E., BEN-LEVY, B., OR, E., ULLRICH, A. & YARDEN, Y. (1991). Oncogenic forms of the neu/HER2 tyrosine kinase are permanently coupled to phospholipase C γ. *EMBO J.*, **10**, 2077–2086.

PIERCE, J.H., ARNSTEIN, P., DIMARCO, E., ARTRIP, J., KRAUS, M.H., LONARDO, F., DI FIORE, P.P. & AARONSON, S.A. (1991). Oncogenic potential of *erb*B-2 in human mammary epithelial cells. *Oncogene*, **6**, 1189–1194.

PRESS, M.F., CORDON-CARDO, C. & SLAMON, D.J. (1990). Expression of the *HER-2/neu* proto-oncogene product in normal human adult and fetal tissue. *Oncogene*, **5**, 953–962.

SEGATTO, O., KING, C.R., PIERCE, J.H., DI FIORE, P.P. & AARONSON, S.A. (1988). Different structural alterations upregulate *in vitro* tyrosine kinase activity and transforming potency of the *erb*B-2 gene. *Mol. Cell Biol.*, **8**, 5570–5574.

SCHECHTER, A.L., STERN, D.F., VAIDYANATHAN, L., DECKER, S.J., DREBIN, J.A., GREENE, M.I. & WEINBERG, R.A. (1984). The *neu* oncogene: an *erb*B-related gene encoding a 185,000-$M_r$ tumour antigen. *Nature*, **312**, 513–516.

SLAMON, D.J., CLARK, G.M., WONG, S.G., LEVIN, W.J., ULLRICH, A. & MCGUIRE, W.L. (1987). Human breast cancer: correlation of relapse and survival with amplification of the *HER2/neu* oncogene. *Science*, **235**, 177–182.

SLAMON, D.J., GODOLPHIN, W., JONES, L.A., HOLT, J.A., WONG, S.G., KEITH, D.E., LEVIN, W.J., STUART, S.G., UDOVE, J., ULLRICH, A. & PRESS, M.F. (1989). Studies of the *HER2/neu* proto-oncogene in human breast and ovarian cancer. *Science*, **244**, 707–712.

STANCOVSKI, I., HURWITZ, E., LEITNER, O., ULLRICH, A., YARDEN, Y. & SELA, M. (1991). Mechanistic aspects of the opposing effects of monoclonal antibodies to the *erb*B-2 receptor on tumor growth. *Proc. Natl Acad. Sci. USA*, **88**, 8691–8695.

SUDA, Y., AIZAWA, S., FURUTA, Y., YAGI, T., IKAWA, Y., SAITOH, K., YAMADA, Y., TOYOSHIMA, K. & YAMAMOTO, T. (1990). Induction of a variety of tumors by c-*erb*B-2 and clonal nature of lymphomas even with the mutated gene (Val659—Glu659). *EMBO J.*, **9**, 181–190.

VAN DE VIJVER, M., VAN DE BERSSELAAR, R., DEVILEE, P., CORNELISSE, C., PETERSE, J. & NUSSE, R. (1987). Amplification of the *neu* (c-*erb*B-2) oncogene in human mammary tumors is relatively frequent and is often accompanied by amplification of the linked c-*erb*A oncogene. *Mol. Cell Biol.*, **7**, 2019–2023.

VARLEY, J.M., SWALLOW, J.E., BRAMMER, W.J., WHITTAKER, J.L. & WALKER, R. (1987). Alterations in either c-*erb*B-2 (*neu*) or c-*myc* proto-oncogenes in breast carcinomas correlate with poor short-term prognosis. *Oncogene*, **1**, 423–430.

WELS, W., HARWERTH, I.-M., ZWICKL, M., HARDMAN, N., GRONER, B. & HYNES, N.E. (1992*a*). Construction, bacterial expression and characterization of a bifunctional single-chain antibody-phosphatase fusion protein targeted to the human *erb*B-2 receptor. *Biotechnology*, **10**, 1128–1132.

WELS, W., HARWERTH, I.-M., MÜLLER, M., GRONER, B. & HYNES, N.E. (1992*b*). Selective inhibition of tumor cell growth by a recombinant single-chain antibody-toxin specific for the *erb*B-2 receptor. *Cancer Res.*, **52**, 6310–6317.

WRIGHT, C., ANGUS, B., NICHOLSON, S., SAINSBURY, J.R.C., CAIRNS, J., GULLICK, W.J., KELLY, P., HARRIS, A.L. & HORNE, C.H.W. (1989). Expression of c-*erb*B-2 oncoprotein: a prognostic indicator in human breast cancer. *Cancer Res.*, **49**, 2087–2090.

YAMAMOTO, T., IKAWA, S., AKIYAMA, T., SEMBA, K., NOMURA, N., MIYAJIMA, N., SAITO, T. & TOYOSHIMA, K. (1986). Similarity of protein encoded by the human c-*erb*B2 gene to epidermal growth factor receptor. *Nature*, **319**, 230–234.

YARDEN, Y. (1990). Agonistic antibodies stimulate the kinase encoded by the *neu* protooncogene in living cells but the oncogenic mutant is constitutively active. *Proc. Natl Acad. Sci. USA*, **87**, 2569–2573.